# Role of Alpha-Synuclein in Frontotemporal Dementia: Narrative Review

**DOI:** 10.3390/cells15050470

**Published:** 2026-03-05

**Authors:** Anastasia Bougea

**Affiliations:** 1st Department of Neurology, Medical School, National and Kapodistrian University of Athens, 11528 Athens, Greece; abougea@med.uoa.gr; Tel.: +30-21-0728-9405

**Keywords:** frontotemporal dementia, alpha-synuclein, FTLD-synuclein, proteinopathy, neurodegeneration, co-pathology, tau, TDP-43, cross-seeding, biomarkers

## Abstract

**Highlights:**

**What are the main findings?**

- Alpha-synuclein cross-seeds with Tau and TDP-43, accelerating neurotoxicity and neuronal loss in FTD.
- Secondary alpha-synuclein pathology predicts poor prognosis, rapid executive decline, and shorter survival times.

**What are the implications of the main findings?**

- Undetected alpha-synuclein may obscure potential trial benefits, necessitating biomarker screening for accurate patient stratification.
- Future therapies must adopt multi-target cocktail approaches to neutralize synergistic protein toxicity simultaneously.

**Abstract:**

Background: Frontotemporal dementia (FTD) is traditionally classified based on the accumulation of either tau or TDP-43 proteins; however, the presence of alpha-synuclein (α-Syn) in these patients is increasingly recognized as a critical factor driving disease progression. Methods: A comprehensive narrative review of recent clinical, neuropathological, and biochemical studies was conducted, focusing on cases of FTLD-synuclein and the occurrence of alpha-syn as a co-pathology in more common FTD variants. Results: Current evidence indicates that α-syn often co-aggregates with tau and TDP-43 via “cross-seeding” mechanisms, significantly accelerating neuronal loss and contributing to clinical heterogeneity. Although FTLD-synuclein is a rare, distinct subtype that mimics atypical multiple system atrophy, secondary α-syn pathology is common and strongly correlates with rapid cognitive decline. Furthermore, existing diagnostic biomarkers typically fail to detect this pathological overlap, which may explain the limited efficacy in protein-specific clinical trials. Conclusions: α-Syn is a major, yet under-recognized, catalyst of neurodegeneration within the FTD spectrum. The findings emphasize the need for future therapeutic and diagnostic strategies to adopt multi-target approaches, addressing the synergistic toxicity of multiple protein aggregates rather than isolating single protein in isolation.

## 1. Introduction

Frontotemporal dementia (FTD) encompasses a complex spectrum of neurodegenerative disorders characterized by progressive deficits in behavior, executive function, and language [1]. It is important to distinguish the clinical syndrome of FTD from its underlying neuropathology, termed frontotemporal lobar degeneration (FTLD). Historically, the neuropathological classification of FTD has relied on the accumulation of specific protein aggregates, predominantly the microtubule-associated protein tau (FTLD-Tau) or the TAR DNA-binding protein 43 (FTLD-TDP) [2]. These proteinopathies have served as the foundational framework for categorization and therapeutic targeting, establishing a binary model that informs current clinical trials. However, emerging neuropathological data suggest that this rigid classification may be inadequate for capturing the full biological reality of the disease [3].

The presence of alpha-synuclein (α-Syn) in patients clinically diagnosed with FTD is increasingly recognized as a pivotal factor in disease progression, challenging the traditional boundaries between FTD and synucleinopathies. Although α-Syn is canonically associated with Lewy Body Dementia (LBD) and Parkinson’s disease (PD), its detection in FTD indicates a significant, yet poorly understood, overlap in neurodegenerative pathways [4,5]. This overlap manifests in two forms: as a primary pathology in the rare FTLD-synuclein subtype, or, more frequently, as a secondary co-pathology alongside Tau or TDP-43 [6]. Recent post-mortem analyses reveal that this co-occurrence is rarely coincidental and is often synergistic [7].

The “pure” proteinopathy model struggles to account for the substantial heterogeneity in clinical phenotypes and rates of decline observed among patients sharing the same primary diagnosis [8]. Mounting evidence demonstrates that the presence of α-Syn accelerates neurodegeneration through “cross-seeding” mechanisms, effectively acting as a catalyst that exacerbates the toxicity of the primary protein aggregate [9]. Despite this, current diagnostic biomarkers and therapeutic strategies remain largely “protein-exclusive,” potentially ignoring a primary driver of neuronal loss in a significant subset of FTD [10].

This narrative review aims to clarify the role of α-Syn within the FTD spectrum, advocating for a shift in the focus from single-protein models to an understanding of complex, mixed proteinopathies. We explore α-Syn not merely as a comorbid bystander, but as an active driver of neurodegeneration. We also highlight the hypothesis that mixed proteinopathies are the rule rather than the exception in aggressive FTD cases, necessitating a critical re-evaluation of how these disorders are defined and treated. Finally, we conclude that α-Syn is a major, under-recognized contributor to the FTD pathological landscape, demanding immediate integration into the design of next-generation diagnostic and therapeutic frameworks.

## 2. Materials and Methods

### 2.1. Search Strategy and Data Sources

A comprehensive narrative review of the literature was conducted to evaluate the role of α-Syn within the FTD spectrum. A total of 850 records were initially identified through systematic searches across PubMed/MEDLINE, Scopus, and Web of Science, alongside manual retrieval of seminal papers. After the removal of duplicate records, 600 unique articles remained. These records were then screened by title and abstract for relevance to the study objectives, leading to the exclusion of 480 articles that did not meet the scope of this review. The remaining 120 full-text articles were rigorously evaluated against the predefined inclusion and exclusion criteria. Following this full-text assessment, a final total of 51 studies were deemed eligible and were qualitatively synthesized to construct the narrative arguments presented in this review. To ensure the inclusion of the most current evidence regarding emerging neuropathological subtypes, the search was limited to articles published between 1 January 2015 and October 2025. However, seminal papers describing the initial characterization of FTLD-synuclein prior to this period were also manually retrieved to provide historical context.

The search strategy utilized a combination of Medical Subject Headings (MeSH) and free-text terms relevant to the disease spectrum and specific proteinopathies. Key search terms included: “Frontotemporal dementia” OR “FTD” OR “Frontotemporal lobar degeneration” OR “FTLD” AND “alpha-synuclein” OR “α-Syn” OR “Lewy bodies” OR “FTLD-synuclein”. Additional targeted searches were conducted to identify mechanisms of protein interaction using terms such as “co-pathology,” “cross-seeding,” “mixed proteinopathy,” “Tau,” and “*TDP-43*”.

### 2.2. Inclusion and Exclusion Criteria

The selection of studies was guided by strict inclusion criteria to focus on the specific interplay between α-Syn and FTD pathologies. Studies were included if they:

1. Reported original data on neuropathologically confirmed cases of FTLD-synuclein (a rare subtype distinct from classic Lewy Body Dementia).
2. Investigated the prevalence or mechanism of alpha-syn co-pathology in patients with primary FTLD-Tau or FTLD-TDP variants.
3. Provided biochemical evidence of protein interactions (e.g., cross-seeding assays) or clinical-pathological correlations involving mixed pathology.

Review articles, editorials, and conference abstracts lacking peer-reviewed full text were generally excluded, except where they offered unique theoretical frameworks regarding biomarkers or clinical trial design. Only articles published in the English language were considered.

### 2.3. Data Extraction and Synthesis

Retrieved articles were screened by title and abstract for relevance. Full-text articles were then evaluated to categorize findings into three main domains as outlined in the study objectives: (1) clinical characteristics of FTLD-synuclein; (2) molecular mechanisms of α-Syn cross-seeding with Tau and TDP-43; and (3) the impact of secondary alpha-synucleinopathy on diagnostic biomarkers and clinical progression. Data regarding patient demographics, protein aggregation patterns, and clinical outcomes were qualitatively synthesized to construct the narrative arguments presented in Section 3.

## 3. Results

### 3.1. Alpha-Synuclein Co-Aggregation and Cross-Seeding Mechanisms

**Figure 1:**
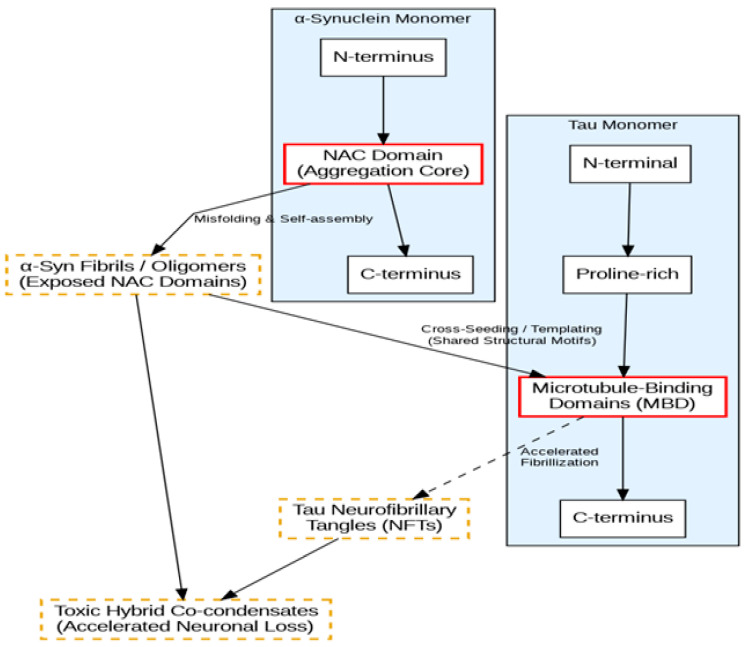
Schematic of α-Synuclein and Tau Cross-Seeding Mechanism. This diagram illustrates the molecular basis for the synergistic toxicity observed in mixed FTD proteinopathies. The central event in the cross-seeding process is driven by interactions between shared structural motifs. Specifically, misfolded α-synuclein fibrils expose their highly amyloidogenic Non-Amyloid Component (NAC) domain, which acts as a pathological template. This exposed NAC domain directly interacts with the Microtubule-Binding Domains (MBD) of Tau monomers. This cross-seeding lowers the thermodynamic barrier for Tau aggregation, driving the accelerated formation of neurofibrillary tangles and highly toxic hybrid co-condensates that overwhelm cellular proteostasis. **Light Blue Backgrounds:** Group the monomeric structures (α-Synuclein Monomer and Tau Monomer) and their constituent domains. **White Boxes with Standard Black Borders:** Represent the standard constituent domains of the native proteins (N-terminus, C-terminus, Proline-rich). **White Boxes with Thick Red Borders:** Highlight the critical interactive domains (NAC Domain and MBD) that serve as the primary aggregation cores. **White Boxes with Dashed Goldenrod Borders:** Indicate misfolded, pathological aggregate states (α-Syn Fibrils/Oligomers, Tau NFTs, and Toxic Hybrid Co-condensates). **Solid Arrows:** Illustrate primary, direct processes such as structural assembly, misfolding, and direct cross-seeding/templating interactions. **Dashed Arrow:** Indicates the secondary, downstream accelerated pathological process (Accelerated Fibrillization) that is triggered by the cross-seeding event.

Before examining the specific role of α-Syn, it is essential to contextualize the broader phenomenon of protein co-aggregation in neurodegenerative diseases [11]. Historically viewed as single-protein disorders, many neurodegenerative conditions are now recognized as complex mixed proteinopathies [11,12]. For example, the co-aggregation of *TDP-43* with other pathogenic proteins, such as Tau and Amyloid-β, is frequently observed not only in FTD and Amyotrophic Lateral Sclerosis (ALS) but also in Alzheimer’s disease and limbic-predominant age-related TDP-43 encephalopathy (LATE) [11,13]. These widespread co-pathologies suggest that the failure of cellular proteostasis often triggers a cascading vulnerability, where the aggregation of one primary amyloidogenic protein accelerates the misfolding of others through shared seeding mechanisms or the overwhelming of clearance pathways [14]. Within this broader landscape of interacting proteinopathies, emerging evidence indicates that similar, synergistic mechanisms may actively operate within the FTD spectrum regarding α-Syn [12]. Recent biochemical studies have elucidated the molecular underpinnings of mixed proteinopathies. While the amyloidogenic properties of α-Syn and its capacity for “cross-seeding” have been extensively characterized in experimental models of PD and LBD [5,15], emerging evidence indicates that similar, synergistic mechanisms may actively operate within the FTD spectrum. In vitro and in vivo models extrapolated from these classic synucleinopathies demonstrate that the misfolded template of one protein can accelerate the aggregation of another. Specifically, α-Syn fibrils have been shown to induce the fibrillization of Tau monomers into neurofibrillary tangles (NFTs)—a synergy that appears more toxic to neurons than either aggregate in isolation [9]. This interaction is facilitated by shared structural motifs in the microtubule-binding domains of Tau and the non-amyloid component (NAC) of α-Syn [16]. In vitro cross-seeding assays have explicitly demonstrated the dynamics of this synergistic toxicity. Studies utilizing fibrillization assays and transgenic models reveal that α-Syn fibrils can act as a primary nucleus to initiate and accelerate Tau aggregation into neurofibrillary tangles. This process results in the formation of highly cytotoxic “hybrid” structures that are significantly more detrimental to cellular survival than single-protein aggregates. Conversely, the interaction is bidirectional; Tau aggregates can also serve as templates that lower the thermodynamic energy barrier for α-Syn, actively promoting its prion-like propagation and spreading throughout neuronal networks (Figure 1).

Beyond Tau, recent biochemical investigations have uncovered profound synergistic interactions between α-Syn and TDP-43. Specifically, the prion-like C-terminal domain of TDP-43 has been shown to interact directly with α-Syn, generating neurotoxic hybrid fibrils that severely exacerbate cellular dysfunction. These interactions are heavily influenced by liquid–liquid phase separation (LLPS), a process where direct contact between TDP-43 and other aggregation-prone proteins promotes rapid co-condensation [17,18,19]. Furthermore, single N-terminal modifications, such as phosphomimetics, can disrupt normal TDP-43 polymerization and phase separation, contributing to the hardening of these hybrid condensates into insoluble, highly toxic inclusions, leading to accelerated synaptic dysfunction and cell death [15,20].

### 3.2. FTLD-Synuclein: A Distinct Subtype

**Figure 2:**
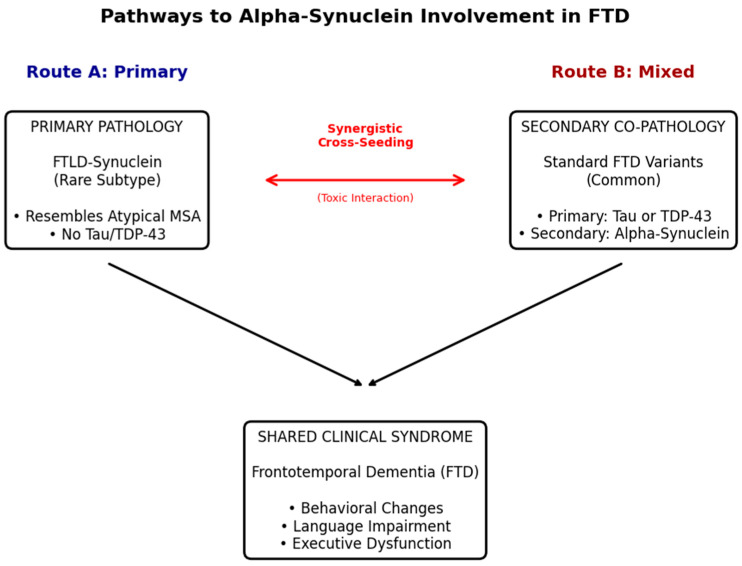
The Dual Role of Alpha-Synuclein in the FTD Spectrum. This schematic illustrates the two primary pathways by which alpha-synuclein (α-Syn) contributes to Frontotemporal Dementia (FTD). (**Left**) Represents FTLD-synuclein, a distinct, rare subtype where α-syn is the primary driver. These cases often clinically resemble atypical Multiple System Atrophy (MSA) with predominant frontotemporal atrophy, distinct from classic Lewy Body Dementia. (**Right**) Represents the more common scenario where α-syn accumulates as a secondary co-pathology alongside Tau or TDP-43. This interaction is driven by “cross-seeding” mechanisms (center arrows), where toxic synergism between proteins leads to accelerated neuronal loss. (**Bottom**) Both pathways converge on a more aggressive clinical prognosis, characterized by rapid cognitive decline compared to single-protein pathologies.

While α-Syn is often a secondary pathology, a distinct subset of patients presents with primary alpha-synucleinopathy that mimics the clinical phenotype of FTD, a condition increasingly referred to as FTLD-synuclein [21,22,23,24]. Neuropathological studies have identified cases initially diagnosed as behavioral variant FTD (bvFTD) or progressive non-fluent aphasia (PNFA) which, upon autopsy, lacked Tau or TDP-43 pathology but exhibited severe alpha-synuclein neuronal inclusions [23,25,26]. Unlike classic LBD, these cases typically spare the neocortex from widespread Lewy bodies and instead show heavy burden in the anteromedial temporal lobe and limbic structures [27]. Crucially, these cases often resemble “atypical multiple system atrophy” (MSA); however, they lack the profound autonomic dysfunction and glial cytoplasmic inclusions (GCIs) characteristic of typical MSA [23]. This dissociation suggests that FTLD-synuclein represents a unique clinicopathological entity where the regional distribution of α-Syn shifts towards frontotemporal networks, driving a cognitive-behavioral phenotype rather than a motor-autonomic one (Figure 2) [28].

### 3.3. Secondary Alpha-Syn Pathology and Clinical Progression

Secondary alpha-syn pathology is frequently observed in cases primarily defined by FTLD-Tau or FTLD-TDP, and its presence is a robust predictor of poor prognosis [3,29,30]. Large-scale clinicopathological correlations indicate that up to 20–30% of patients with primary FTLD-Tau harbor concomitant alpha-syn aggregates [31,32,33,34]. In these mixed-pathology cohorts, the clinical trajectory is markedly different from “pure” FTLD forms. Patients with co-pathology exhibit a more rapid decline in executive function and shorter survival times from symptom onset [35,36]. Furthermore, the presence of α-Syn in FTLD-TDP cases has been linked to greater cortical atrophy and an increased incidence of Parkinsonian motor features that are otherwise atypical for TDP-43 proteinopathies [7]. This data supports the hypothesis that secondary alpha-synucleinopathy acts as a “disease modifier,” lowering the threshold for neurodegeneration and exacerbating the clinical severity of the primary underlying proteinopathy [36] (Table 1).

Furthermore, the presence of α-Syn in FTLD-TDP cases has been linked to greater cortical atrophy and an increased incidence of Parkinsonian motor features that are otherwise atypical for TDP-43 proteinopathies [37,38]. This specific co-occurrence supports the hypothesis that secondary alpha-synucleinopathy acts as a “disease modifier,” effectively lowering the threshold for neurodegeneration and exacerbating the clinical severity of the primary underlying proteinopathy.

### 3.4. Biomarker Limitations and Diagnostic Challenges

The detection of α-Syn co-pathology in FTD remains a significant diagnostic gap. Current fluid biomarkers, such as cerebrospinal fluid (CSF) levels of total and phosphorylated α-Syn, lack the sensitivity to distinguish FTD cases with secondary α-Syn from those without [39]. While the newly developed α-Syn Seed Amplification Assays (α-SAA) have revolutionized the diagnosis of PD and LBD with sensitivities exceeding 90%, their utility in identifying the specific strain of α-Syn present in FTLD-synuclein or mixed FTD cases is not yet fully established [40]. Preliminary data suggests that the alpha-syn aggregates in FTLD variants may exhibit distinct conformational properties that result in lower seeding activity in standard SAA protocols [41,42,43,44]. Consequently, many patients with mixed pathology test “negative” for α-Syn in vivo, leading to their inclusion in pure Tau or TDP-43 clinical trials. This diagnostic blind spot potentially confounds trial results, as the unchecked synucleinopathy may continue to drive neurodegeneration even if the primary protein target is successfully engaged [39,45].

### 3.5. Genetic Intersections in Mixed Proteinopathies

The structural and clinical overlap between FTD and synucleinopathies is further reinforced by genetic evidence. Pathogenic mutations canonically associated with one disease spectrum often manifest with co-pathologies characteristic of the other. For instance, individuals carrying the p.A53T mutation in the α-synuclein gene (SNCA) can present clinically with a frontotemporal dementia phenotype rather than classical Parkinsonism [21]. Conversely, patients harboring pathogenic variants in the MAPT gene, which typically strictly causes FTLD-Tau, have been documented presenting with clinical features mimicking DLB [38]. Furthermore, FTD cases driven by TBK1 or C9orf72 mutations are increasingly shown to harbor concurrent α-Syn alongside primary Tau or TDP-43 pathologies, highlighting a shared, genetically driven vulnerability in neuronal proteostasis [10,34].

## 4. Discussion

The findings of this review indicate that future therapeutic and diagnostic strategies must transition toward multi-target approaches. The traditional “silver bullet” paradigm, which targets a single protein in isolation, appears inadequate for a substantial subset of FTD patients.

### 4.1. The Synergistic Effect

The “cross-seeding” mechanisms discussed above highlight a synergistic effect wherein the toxicity of combined aggregates exceeds the sum of their individual parts. This interaction suggests that the presence of α-Syn may alter the conformational strains of Tau or TDP-43, potentially creating “hybrid” strains characterized by enhanced seeding capacity and increased resistance to cellular clearance mechanisms [46,47]. Moreover, this phenomenon points to a mechanism of prion-like propagation, where α-Syn fibrils serve as templates that lower the thermodynamic energy barrier for Tau aggregation [48].

This synergy offers a plausible explanation for the rapid clinical deterioration observed in mixed-pathology cases, as the cellular proteostatic machinery becomes overwhelmed by the simultaneous burden of distinct amyloidogenic substrates. Consequently, the failure of these proteostatic systems may be driven not solely by the total protein burden, but rather by the complex interplay between distinct misfolded species—a dynamic that current single-protein models fail to adequately capture (Table 2).

### 4.2. Implications for Clinical Trials

We hypothesize that the lack of efficacy observed in several protein-specific clinical trials might be partially linked to α-Syn acting as an under-recognized driver of neurodegeneration. The failure to screen for α-Syn co-pathology in FTD trials introduces significant confounding variables. While many trials have historically proceeded without such screening, the “noise” generated by undetected, secondary synucleinopathy may obscure potential therapeutic benefits in the target population [49]. For instance, if a therapeutic agent successfully clears Tau but leaves α-Syn aggregates intact, it is highly plausible that the “cross-seeding” engine remains active, thereby perpetuating neurodegeneration [5].

Consequently, future trial designs must prioritize the routine implementation of α-Syn biomarkers, such as SAA, to identify co-pathology prior to enrollment. Concurrently, rigorous patient stratification is necessary, effectively grouping individuals based on their “proteotype”—distinguishing mixed from pure pathology—rather than relying solely on their clinical phenotype [50]. These structural changes must be supported by the development of companion diagnostics capable of quantifying total amyloid burden, thereby enabling the deployment of precision medicine strategies that match therapeutic cocktails to the specific molecular profile of the patient.

To address this, future trial designs must prioritize the routine implementation of sensitive α-Syn biomarkers to identify co-pathology prior to enrollment, effectively grouping individuals based on their “proteotype” rather than just their clinical phenotype [51]. This structural shift is essential for the advancement of “cocktail” therapies that can address the synergistic effects of multiple protein aggregates simultaneously [52]. Without such stratification, the unchecked synucleinopathy will continue to drive neuronal loss even if the primary target is engaged, potentially explaining why single-target interventions have historically failed to alter the disease course in heterogeneous FTD populations [51]. The field must move toward precision medicine strategies that match therapeutic combinations to the specific molecular profile of the patient, accounting for total amyloid burden rather than treating proteins in isolation. Without this stratification, unchecked synucleinopathy will likely continue to drive neuronal loss even if the primary target is successfully engaged, potentially explaining why single-target interventions have historically failed to alter the disease course in heterogeneous FTD populations.

To actively address this synergistic toxicity, the field must advance towards precision therapies. Quantitative systems pharmacology models evaluating the clinical failure of single-target anti-tau or anti-synuclein antibodies have underscored the mathematical necessity of this paradigm shift. By employing multi-target “cocktail” strategies similar to modern oncology, combined immunotherapies targeting both α-Syn and the primary protein (e.g., Tau) could effectively neutralize the “cross-seeding” engine.

### 4.3. Redefining the Neuropathological Spectrum

Finally, the accumulating evidence regarding FTLD-synuclein and widespread co-pathology compels a re-evaluation of the nosological boundaries between FTD and LBD. The rigid separation of these entities often forces patients into single-protein categories (e.g., FTLD-Tau vs. FTLD-TDP), ignoring the biological reality of mixed proteinopathies [37,53]. Rather than viewing FTLD-synuclein as a rare outlier, it may be more accurate to view these disorders as existing on a phenotypic continuum. This perspective advocates for a shift toward a biological definition of FTD, where diagnosis is based on the specific combination of protein aggregates present [54]. Such a shift is essential for the advancement of “cocktail” therapies—similar to those used in oncology—that can address the synergistic effects of multiple protein aggregates simultaneously.

This blur between traditionally distinct categories advocates for a shift toward a strictly biological definition of FTD. Characterizing patients by their specific “proteotype”—which accounts for the exact combination of protein aggregates present—is essential [55]. Such comprehensive profiling is a fundamental prerequisite for precision medicine, enabling the accurate deployment of the aforementioned therapeutic cocktails capable of neutralizing the synergistic effects of multiple protein aggregates simultaneously.

## 5. Conclusions

α-Syn emerges as a major, yet under-recognized, potential driver of neurodegeneration within the FTD spectrum. While FTD is traditionally defined by Tau and TDP-43 proteinopathies, the accumulating evidence for FTLD-synuclein and secondary α-Syn co-pathology challenges the rigid binary classification of the disease. The biochemical reality of “cross-seeding”—where α-Syn may actively catalyze the toxicity of Tau and TDP-43—provides a compelling hypothesis for the accelerated neuronal loss observed in mixed-pathology cases compared to single-protein conditions. Furthermore, this synergistic interaction may contribute to the failure of previous protein-specific trials, as unchecked synucleinopathy could continue to drive disease progression even when the primary therapeutic target is successfully engaged.

However, significant gaps remain, particularly regarding the validation of longitudinal biomarkers and the in vivo characterization of distinct α-Syn strains specific to FTD. Current fluid assays and standard seed amplification protocols must be further refined to accurately detect FTD-specific co-pathologies. Despite these challenges, there is cautious optimism for the future. To meaningfully improve patient outcomes, the field must gradually transition away from “pure” pathology models and embrace a broader biological definition of FTD based on comprehensive proteotypes. Future success will heavily depend on developing robust multi-target diagnostic frameworks and adopting “cocktail” therapies that address these complex, mixed proteinopathies simultaneously.

## Figures and Tables

**Table 1:** Clinicopathological Correlations: Impact of Primary and Secondary Alpha-Synuclein Pathology on Clinical Phenotypes in the FTD Spectrum.

Protein Aggregate Classification	Associated Clinical Diagnosis	Key Clinical Features & Phenotype	Prognostic Impact
FTLD-Tau (Pure)	bvFTD, PNFA	Standard presentation of behavioral, executive, and language deficits without significant motor features initially.	Standard progression rates associated with classic FTD.
FTLD-TDP (Pure)	bvFTD, Semantic Dementia	Characterized by deficits in behavior and language; typically lacks significant Parkinsonian features in pure forms.	Standard progression rates; less aggressive than mixed cases.
FTLD-Synuclein (Primary/Distinct Subtype)	Mimics bvFTD or PNFA	Atypical MSA Presentation: Resembles “atypical multiple system atrophy” but lacks profound autonomic dysfunction. Brain Distribution: High burden in anteromedial temporal lobe/limbic structures rather than neocortex.	distinct clinicopathological entity; progression mimics FTD rather than motor-autonomic disorders.
Mixed: Tau + Alpha-Syn (Secondary Co-pathology)	Primary FTLD-Tau variant	Executive Deficit: Patients exhibit a more rapid decline in executive function compared to pure Tau cases.	Aggressive: Significantly shorter survival times from symptom onset.
Mixed: TDP-43 + Alpha-Syn (Secondary Co-pathology)	Primary FTLD-TDP variant	Motor Features: Increased incidence of Parkinsonian motor features (atypical for pure TDP-43). Atrophy: Linked to greater cortical atrophy.	Accelerated: Evidence suggests it acts as a “disease modifier” lowering the threshold for neurodegeneration.

bvFTD: Behavioral Variant Frontotemporal Dementia; PNFA: Progressive Non-Fluent Aphasia; FTLD: Frontotemporal Lobar Degeneration; MSA: Multiple System Atrophy; TDP-43: TAR DNA-binding protein 43. The “Mixed” categories refer to cases where α-Syn is present as a secondary pathology alongside the primary protein aggregate (Tau or TDP-43).

**Table 2:** Protein Interactions and α-Syn Co-pathology in FTD of selected studies.

Study (Author, Year)	Primary Protein Target	Coexistence of α-Syn	Sample Characteristics	Assay/Investigation Type	Main Outcomes
Hu et al. (2017) [9]	Tau Monomers	Cross-Seeding	In vitro/Cell models	In vitro cross-seeding assays	α-Syn fibrils seed Tau aggregation; forming highly cytotoxic structures.
Dhakal et al. (2021) [15]	TDP-43 (Prion-like domain)	Synergistic: Direct interaction with α-Syn	In vitro models	Protein interaction assays	Direct interaction between TDP-43 and α-Syn, forms neurotoxic hybrid fibrils.
Giasson et al. (2003) [16]	Tau Monomers	Synergistic: Synergistic fibrillization	Transgenic mice; In vitro	Fibrillization assays & IHC	α-Syn fibrils initiate Tau aggregation into neurofibrillary tangles (synergistic toxicity).
Pan et al. (2022) [48]	Tau Aggregates	Catalytic: Tau accelerates α-Syn spreading	In vivo models	Spreading/Seeding assays; Immunofluorescence	Tau acts as a template to lower the energy barrier for α-Syn aggregation, promoting prion-like propagation.
Aoki et al. (2015) [23]	None (Tau-Negative)	Primary: α-Syn accumulation	Human Post-mortem (bvFTD/PNFA)	Neuropathological examination	“atypical MSA” as a distinct FTLD subtype (FTLD-synuclein) with frontotemporal distribution
Cullinane et al. (2025) [22]	α-Syn Filaments	Primary: Structural variant	Human tissue-derived filaments	Cryo-EM & Seeding Assay	FTLD-synuclein and typical MSA share identical filament structures despite differing clinical phenotypes.
Kapasi et al. (2017) [35]	Mixed Proteinopathies	Cumulative burden	Human Clinicopathological Cohort	Clinical-Pathological correlation	Multiple proteinopathies result in faster cognitive decline compared to single-protein diseases.
Robinson et al. (2023) [32]	FTLD-Tau Inclusions	Concomitant α-Syn	Large Human Cohort	Statistical correlation	Pathological combinations are frequent and significantly worsen clinical prognosis.
Orrú et al. (2025) [43]	α-Syn Strains	Strain differentiation	Human CSF/Biosamples	SAA	Distinct SAA kinetic profiles in could potentially differentiate between various synuclein strains.
Irwin et al. (2013) [29]	Neurofibrillary Tangles	Convergent: Overlap	Human Post-mortem (PD Dementia)	Comparative Neuropathology	Convergence of α-Syn and Tau pathologies drives dementia, progression (model for mixed FTD).

α-Syn: Alpha-Synuclein; TDP-43: TAR DNA-binding protein 43; bvFTD: Behavioral Variant Frontotemporal Dementia; PNFA: Progressive Non-Fluent Aphasia; FTLD: Frontotemporal Lobar Degeneration; MSA: Multiple System Atrophy; PD: Parkinson’s Disease; IHC: Immunohistochemistry; Cryo-EM: Cryo-Electron Microscopy; SAA: Seed Amplification Assay; CSF: Cerebrospinal Fluid. The column “Primary Protein Target” refers to the specific form of Tau protein (e.g., monomer vs. aggregate) investigated in the respective study.

## Data Availability

No new data were created or analyzed in this study. Data sharing is not applicable to this article.
